# Supplementary material for: Causal Relationship between Aging and Anorexia Nervosa: A White-Matter-Microstructure-Mediated Mendelian Randomization Analysis
Source: Biomedicines. 2024 Aug 16;12(8):1874. doi: 10.3390/biomedicines12081874 (PMC11351342; doi:10.3390/biomedicines12081874)
Supplement: Supplementary file 1 [file biomedicines-12-01874-s001.zip › Supplementary Figure/Figure S4/Singe SNP plot of MR analysis for TL to ubm-b-2124.pdf]

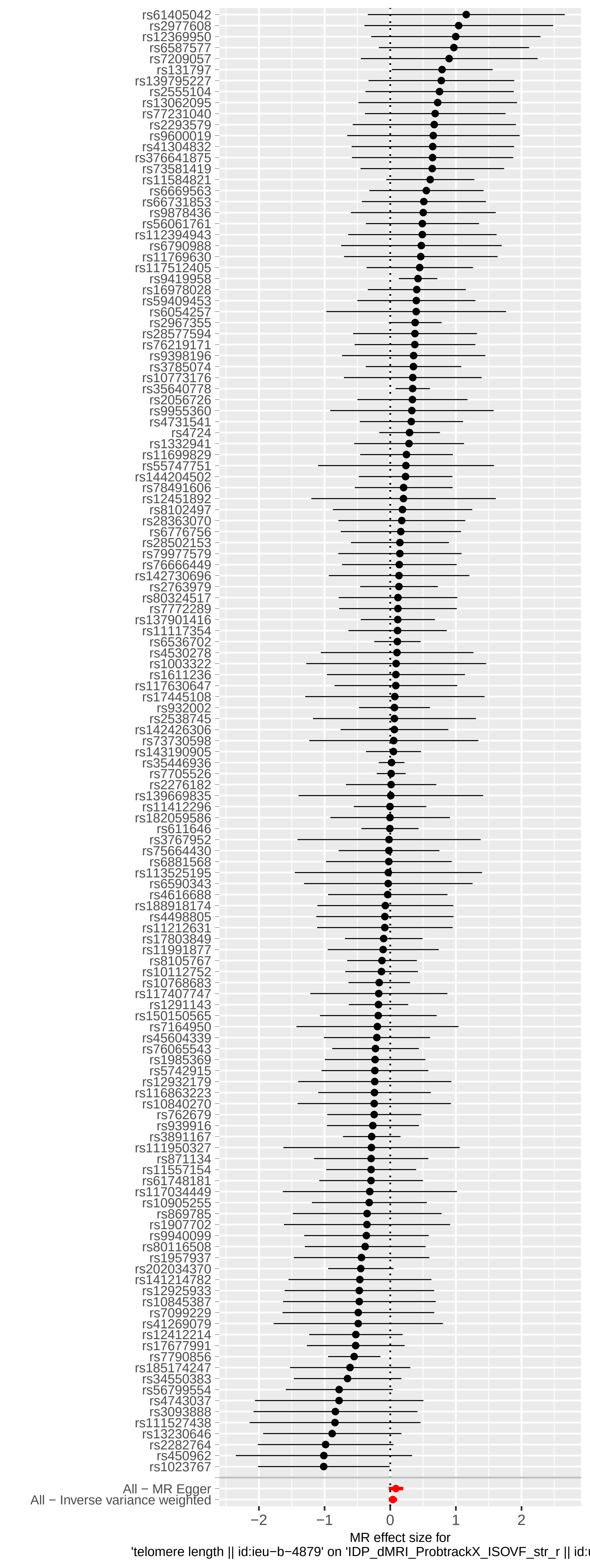
rs61405042
rs2977608
rs12369950
rs6587577
rs7209057
rs131797
rs139795227
rs2555104
rs13062095
rs77231040
rs2293579
rs9600019
rs41304832
rs376641875
rs73581419
rs11584821
rs6669563
rs66731853
rs9878436
rs56061761
rs112394943
rs6790988
rs11769630
rs117512405
rs9419958
rs16978028
rs59409453
rs6054257
rs2967355
rs28577594
rs76219171
rs9398196
rs3785074
rs10773176
rs35640778
rs2056726
rs9955360
rs4731541
rs4724
rs1332941
rs11699829
rs55747751
rs144204502
rs78491606
rs12451892
rs8102497
rs28363070
rs6776756
rs28502153
rs79977579
rs76666449
rs142730696
rs2763979
rs80324517
rs7772289
rs137901416
rs11117354
rs6536702
rs4530278
rs1003322
rs1611236
rs117630647
rs17445108
rs932002
rs2538745
rs142426306
rs73730598
rs143190905
rs35446936
rs7705526
rs2276182
rs139669835
rs11412296
rs182059586
rs611646
rs3767952
rs75664430
rs6881568
rs113525195
rs6590343
rs4616688
rs188918174
rs4498805
rs11212631
rs17803849
rs11991877
rs8105767
rs10112752
rs10768683
rs117407747
rs1291143
rs150150565
rs7164950
rs45604339
rs76065543
rs1985369
rs5742915
rs12932179
rs116863223
rs10840270
rs762679
rs939916
rs3891167
rs111950327
rs871134
rs11557154
rs61748181
rs117034449
rs10905255
rs869785
rs1907702
rs9940099
rs80116508
rs1957937
rs202034370
rs141214782
rs12925933
rs10845387
rs7099229
rs41269079
rs12412214
rs17677991
rs7790856
rs185174247
rs34550383
rs56799554
rs4743037
rs3093888
rs111527438
rs13230646
rs2282764
rs450962
rs1023767
All − MR Egger
All − Inverse variance weighted
−2
−1
0
1
2
MR effect size for
'telomere length || id:ieu−b−4879' on 'IDP_dMRI_ProbtrackX_ISOVF_str_r || id:
